# Red flags for the early detection of spinal infection in back pain patients

**DOI:** 10.1186/s12891-019-2949-6

**Published:** 2019-12-13

**Authors:** Mohamed Yusuf, Laura Finucane, James Selfe

**Affiliations:** 1 Department of Health Professions, Manchester Metropolitan University, Brooks Building, Birley Campus 53 Bonsall Street, Manchester, M15 6GX UK; 2 Manchester Metropolitan Univeristy, Manchester, UK; 3 Sussex MSK Partnership, Brighton, UK

**Keywords:** Back pain, Spinal pain, Serious spinal pathology, Red flags, Spinal infection, Musculoskeletal infection

## Abstract

**Background:**

Red flags are signs and symptoms that are possible indicators of serious spinal pathology. There is limited evidence or guidance on how red flags should be used in practice. Due to the lack of robust evidence for many red flags their use has been questioned. The aim was to conduct a systematic review specifically reporting on studies that evaluated the diagnostic accuracy of red flags for Spinal Infection in patients with low back pain.

**Methods:**

Searches were carried out to identify the literature from inception to March 2019. The databases searched were Medline, CINHAL Plus, Web of Science, Embase, Cochrane, Pedro, OpenGrey and Grey Literature Report. Two reviewers screened article texts, one reviewer extracted data and details of each study, a second reviewer independently checked a random sample of the data extracted.

**Results:**

Forty papers met the eligibility criteria. A total of 2224 cases of spinal infection were identified, of which 1385 (62%) were men and 773 (38%) were women mean age of 55 (± 8) years. In total there were 46 items, 23 determinants and 23 clinical features. Spinal pain (72%) and fever (55%) were the most common clinical features, Diabetes (18%) and IV drug use (9%) were the most occurring determinants. MRI was the most used radiological test and *Staphylococcus aureus* (27%), *Mycobacterium tuberculosis* (12%) were the most common microorganisms detected in cases.

**Conclusion:**

The current evidence surrounding red flags for spinal infection remains small, it was not possible to assess the diagnostic accuracy of red flags for spinal infection, as such, a descriptive review reporting the characteristics of those presenting with spinal infection was carried out. In our review, spinal infection was common in those who had conditions associated with immunosuppression. Additionally, the most frequently reported clinical feature was the classic triad of spinal pain, fever and neurological dysfunction.

This is an Open Access article distributed in accordance with the Creative Commons Attribution Non-Commercial (CC BY-NC 4.0) license, which permits others to distribute, remix, adapt, build upon this work non-commercially, and license their derivative works on different terms, provided the original work is properly cited and the use is non-commercial. See: http://creativecommons.org/licenses/by-nc/4.0/

## Background

Medical doctors and, musculoskeletal practitioners such as Physiotherapists, Osteopaths and Chiropractors have traditionally used red flags to help in the identification of patients with serious spinal pathology. There are 163 individual items that could be considered as red flags; 119 items in the patient history and 44 items in the physical examination (CSP 2007). Clearly, this presents a problem in terms of the practical clinical utility. In addition, there is limited evidence or guidance on how these red flags should be used in practice [1–3]. Due to the lack of robust evidence for many red flags their use has been called into question [4, 5]. However, clinicians still need to decide whether the patient’s problem is suitable for immediate conservative management (keep), or whether the patient needs to be referred for further investigation (refer). Therefore in spite of no consensus in either guidelines or research, red flags are still seen as the most reliable clinical indicator for potential serious pathology and remain fundamental to the assessment process [6].

Although infections of the spine such as extra pulmonary Tuberculosis (TB) are uncommon, they are on the rise [7, 8]. The majority of SI are of insidious onset and commonly there is a prolonged period of time between onset and diagnosis which can create a complex clinical picture as patients can remain relatively healthy until symptoms manifest themselves in the later stages of the disease [9, 10]. Back pain is the most common presenting symptom which can progress to neurological symptoms and if not treated in a timely manner, lead to serious complications such as paralysis, instability of the spine and can ultimately be fatal [11–14].

Spinal infection often typically thought of as having a long prodromal period, however, Sapico and Montgomerie [10] report 30% of patients were diagnosed from three weeks to three months and 20% of patients were diagnosed in less than three weeks. In cases where there is a prolonged prodromal period, it is unsurprising that errors in the diagnosis of SI in back patients are relatively frequent [15]. When it comes to diagnostic errors in primary care, infection emerges as one of the most significant categories along with cancer and cardiovascular disease. The personal and economic consequences of these errors is significant and are a global burden [16]. For example the legal cost of errors in the diagnosis of SI in the National Health Service (NHS) in the UK is considerable. Between 2002 and 2010, SI accounted for 11.6% of all spinal related malpractice litigation, with the average damage costing the NHS £433,296 per case [17]. These figures are likely to rise, as reported by the Medical Protection Society (MPS). The clinical negligence costs over the past five years within the NHS increased by 72% and are projected to rise to £2.6 billion per year by 2022 [18].

Two of the solutions proposed by the World Health Organisation (WHO) to reduce the global burden of diagnostic errors in Primary Care are to improve diagnostic reasoning and optimise diagnostic strategies [19]. As such this review provides the basis for the construction of a robust International evidence-based clinical framework to improve the identification of patients with SI. Errors in the diagnosis of SI are reported to be as a result of two issues, first failure to recognise the relevant red flags, and second failure to consider SI as a potential differential diagnosis [20–23]. It is therefore vital that clinicians are aware of the possible signs and symptoms of SI and the risk of SI that an individual may have. When used, this knowledge will help to raise the index of suspicion and aid in the early identification of SI.

The primary aim of this study was to investigate the existing evidence to support the use of red flags for identifying Spinal Infection. Therefore, we searched the literature and reviewed studies reporting red flags in Spinal Infection patients.

## Methods

This review was registered with the International Prospective Register of Systematic Reviews on 24/11/17 (PROSPERO; http://www.crd.york.ac.uk/prospero, reference: CRD42017081447). The review was conducted in concordance with the Prefered Reporting Items for Systematic Review and Meta-Analysis (PRISMA) guideline [24]. Our apriori aim was to conduct a systematic review specifically reporting on studies that evaluated the diagnostic accuracy of red flags for SI in patients with low back pain. Due to the paucity of studies evaluating the diagnostic accuracy of red flags for SI, unfortunately, it was not possible to conduct a systematic review on the diagnostic accuracy of red flags as initially intended, therefore, a descriptive review reporting on the characteristics of patients with SI was conducted.

### Literature search

We searched the following electronic databases from inception to March 2019: Medline, CINHAL Plus, Web of Science, Embase, Pedro and Cochrane. In addition to this, OpenGrey and Grey Literature Report were also searched. The database searches were accompanied by hand searches of the reference list of included articles and the grey literature. With the help of the University librarian a search strategy was set in place, the three key search terms used were red flag, spinal pain and infection – see *additional file 1* for the search strategy.

### Study selection

Studies investigating red flags in SI were included. More specifically, the eligible studies were study designs and research articles that had primary data, this included diagnostic accuracy studies, cohort studies, case-control studies, and case-series studies. Two reviewers (*MY* & *LF*), independently, screened the eligible papers against the full article eligibility criteria, any disagreements between the two reviewers were resolved by a third reviewer (*JS*). Included studies were studies in the English language that studied adults over the age of 18 who had a spinal infection, this included Bacterial, Viral, Fungal, Prionic or Parasitic infections. Further, studies were included if they contained primary data such as clinical tests, diagnostic tests, history taking and/or physical examination and provided red flags or clinical features for Spinal Infection. Studies were excluded if they were a systematic review or a narrative review.

### Quality appraisal

Once the full eligible papers were selected, the quality of the studies was assessed using the National Heart, Lung and blood institute (NHLBI) Quality Assessment Tools. These widely used assessment tools determine the quality of case-series, case-control and cohort studies.

### Data extraction

One reviewer (*MY*) extracted the data from all the eligible papers. The following data were extracted from each study: study characteristics, participant characteristics, setting and diagnostic methods, red flags (exposures) and spinal infection (outcomes). After the data was extracted, 25% of the data was independently checked by reviewer two (*LF*), as recommended by the 2009 Updated Method Guidelines for Systematic Reviews in the Cochrane Back Review Group [25].

### Data analysis

Patient data from the included studies were analysed descriptively using frequencies and percentages.

### Patient and public involvement

There were no patient or public involvement in any phase of this study, this included the development of the research question, the analysis and the conclusions.

## Results

**Fig. 1:**
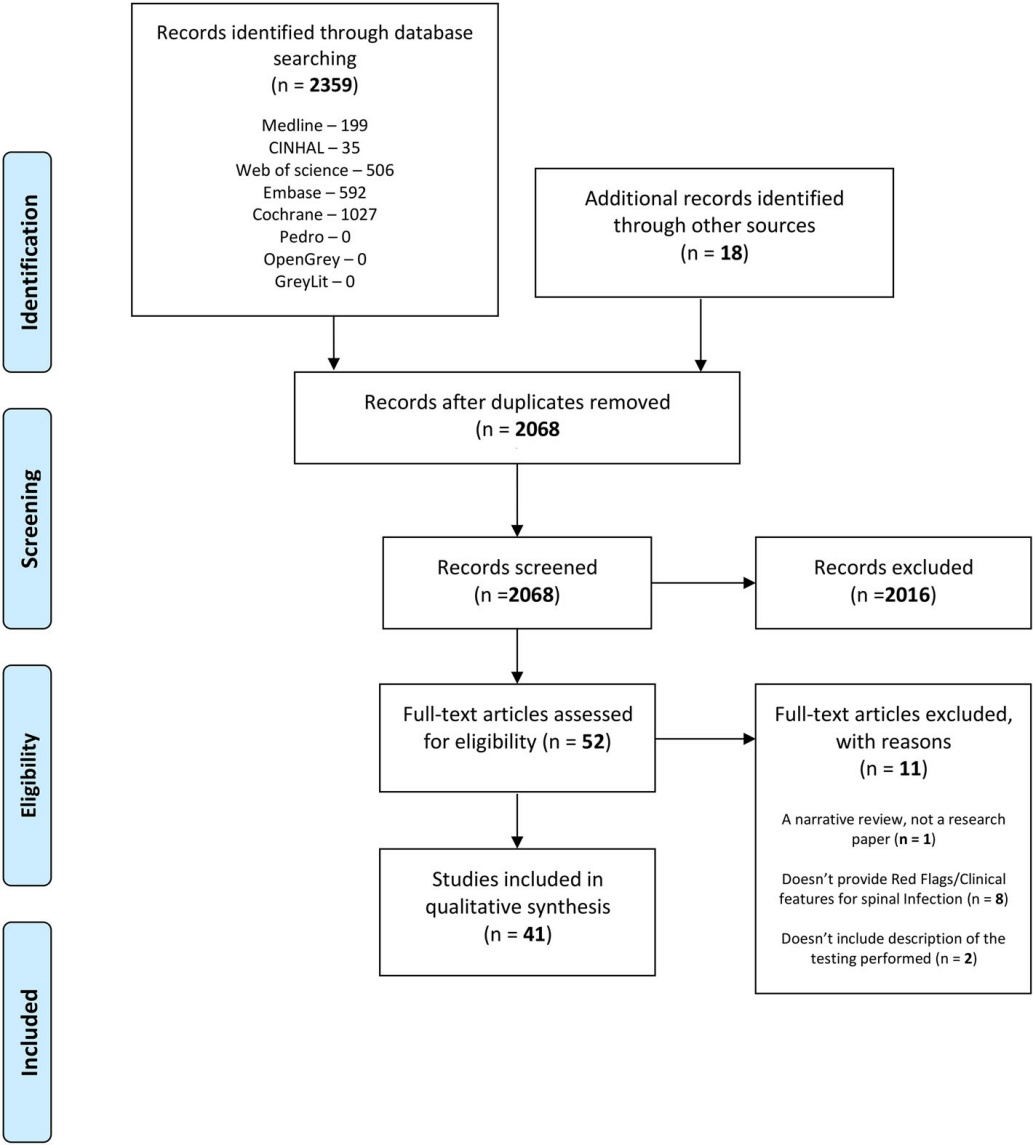
PRISMA 2009 flow diagram

The search strategy yielded 2571 eligible papers, further narrowed down to 2274 papers after duplicates were removed. Once the eligibility criteria for the title and abstract were applied, the total came to 52 papers, of which 18 were from bibliographic searches. See Fig. 1.

### Study selection

After reviewing the 52 eligible papers, 40 papers met the full eligibility criteria as agreed by the two reviewers (see additional file 1). One paper was excluded from the review because it was a narrative review [26], two papers did not include a description of the testing performed [27, 28] and nine papers were excluded because they did not provide data for red flags/clinical features for SI [11, 12, 28–35].

The kappa statistics for the agreement between the two reviewers was *k =* 0.80, this is considered as ‘Very good’ [36, 37]. Reviewer three (*JS*) resolved the disagreement between the first two reviewers, the three discordant papers were included in the study [38–40]. All disagreements were due to criterion three, whether the papers provided clinical features for SI.

### Characteristic of included papers

For a summary of the main characteristics in each paper, see additional file 1. Table 1 presents a summary of the baseline characteristics of the patients in the 40 papers reviewed. A total of 2224 patients were diagnosed, including 1385 male patients (62%) and 773 female patients (38%) with a mean age of 55 years (SD = 8). In total, 14 out of 40 papers did not describe or adequately describe the radiological tests performed. This includes plain film radiography, ultrasound, magnetic resonance imaging scan (MRI), gallium scan, bone scan, computed tomography (CT) scan and myelogram [7, 13, 15, 39, 41–51]. MRI was the most used radiological test with 22 papers using it for investigation. For individual investigations, Plain radiographs and MRI were the most used radiological tests. Bone scans and Gallium scans were the most accurate tests with a positive result of 93 and 90%, respectively. However, ultrasound was the least sensitive test with a positive result of 21%. Out of all studies, 34 (85%) were carried out in High-Income countries and 6 (15%) were carried out in Upper middle-income countries, none were carried out in Lower Middle-Income and Lower Income countries.

**Table 1:** Study Characteristics

Characteristics	Total	Sum of papers
No. patients	2224	40
Gender		39
Male (%)	1385 (62.3)	
Female (%)	773 (37.7)	
Age (mean ± SD) years	55.4 (± 8.2)	35
Data Collection Period (mean ± SD) years	8 (± 4)	40
Diagnostic Tests positive/performed (%)		
Plain Radiograph	599/773 (77.5)	14
MRI	505/632 (80.0)	22
CT Scan	342/395 (86.6)	15
Bone Scan	215/232 (92.7)	11
Gallium Scan	65/72 (90.3)	5
Myelogram	39/46 (84.5)	3
Ultrasound	8/39 (20.5)	3
Laboratory tests*	2322/3867 (60.0)	33
Surgery	93/112 (83.0)	6
World Bank Classification by income (%)		40
High Income (HI)	34 (85.0)	
Upper Middle Income (UMI)	6 (15.0)	
Lower Middle Income (LMI)	0	
Lower Income (LI)	0	

*Tests included are white blood cell count (WBC), C-reactive protein (CRP) and erythrocyte sedimentation rate (ESR), pus culture, blood culture and biopsy

### Red flags

The list of red flags is presented in two groups, determinants and clinical features (Table 2). This decision was made because of the way in which the papers reported this information. Determinants were defined as factors that may play a part in the occurrence of SI, this included comorbidities, social factors and sources of infection that may lead to SI. Within the Dictionary of Epdimeiology, Last et al. define determinant as:

“a collective or individual risk factor (or set of factors) that is causally related to a health condition, outcome, or other defined characteristic. In human health —and, specifically, in diseases of complex etiology—determinants often act jointly in relatively complex and long-term processes. They commonly operate at aggregate (e.g., social, regional, global) levels, as well as at the individual, personal level..” [52].

**Table 2:** Characteristics of SI

Determinants	Total cases Reported (%)	Sum of Papers	Clinical Features	Total cases Reported (%)	Sum of Papers
Morbidities			Systemically Unwell		
Diabetes	399 (17.9)	30	Fever	1225 (55.1)	36
Cancer	112 (5.5)	19	Weight loss/ Anorexia	173 (7.8)	8
Cardiovascular disease	109 (4.9)	6	Rigours	146 (6.6)	3
Renal failure	56 (2.5)	10	Fatigue or weakness	102 (4.6)	6
Liver disease	40 (1.8)	10	Sweats/Night sweats	76 (3.4)	5
Blood pressure dysfunction	40 (1.8)	6	Active infection	54 (2.4)	1
Rheumatoid Arthritis	35 (1.6)	7	Sepsis/Septic Shock	36 (1.6)	2
Social Factors			Pain		
Intravenous drug use	210 (9.4)	15	Spinal Pain	1591 (71.5)	36
Corticosteroid use	72 (3.2)	7	Radiculopathy	214 (9.6)	12
Alcoholism	41 (1.8)	8	Tenderness	116 (6.6)	6
Triggers			Arthralgia	33 (1.5)	3
Surgery	124 (5.6)	10	Myalgia	23 (1.0)	3
Pre-existing Infection	123 (5.5)	15	Sciatic pain	19 (0.9)	2
Immunosuppression	96 (4.3)	8	Neurological Symptoms		
Invasive procedure	42 (1.9)	6	Neurological dysfunction	739 (33.2)	26
Spinal Trauma	32 (1.4)	6	Limb weakness	175 (7.9)	9
Environmental Factors			Para/quadriplegia	51 (2.3)	5
Migrant	70 (3.1)	1	Para/quadriparesis	46 (2.1)	4
Occupational exposure	42 (1.9)	3	Paralysis	46 (2.1)	2
Lived in rural area	28 (1.3)	1	Urological Symptoms		
Born in TB endemic country	18 (0.8)	1	Bladder/bowl dysfunction	62 (2.8)	4
Behavioural Factors			Urinary incontinence	21 (0.9)	2
Ingestion of unpasteurised dairy product	61 (2.7)	3	Organ Involvement		
Contact with infected animals	29 (1.3)	1	Hepatosplenomegaly	21 (0.9)	4
Predisposing Factors			Miscellaneous		
None & Miscellaneous	116 (5.2)	8	Constitutional Symptoms	199 (8.9)	4
History of TB	19 (0.9)	1	Spinal deformity	30 (1.3)	1

**Table 3:** Causative Pathogens

Microbiology	Total (%)	Sum of papers
Bacterial	1665 (74.9)	37
*Staphylococcus aureus*	598 (26.9)	27
*Mycobacterium tuberculosis*	262 (11.8)	13
*Brucella*	210 (9.4)	9
*Streptococcus*	122 (5.5)	19
*Escherichia coli*	86 (3.9)	20
*Methicillin-resistant Staphylococcus aureus*	81 (3.6)	7
*Staphylococcus epidermitis*	53 (2.4)	9
*Psuedomonas*	32 (1.4)	13
*Methicillin-susceptible Staphylococcus aureus*	25 (1.1)	2
*Proteus*	11 (0.5)	5
*Salmonella*	10 (0.4)	6
*Staphylococcus, coagulase negative*	10 (0.4)	4
Other	165 (7.33)	16
Fungal		
*Candida*	12 (0.5)	5
Mixed-growth	27 (1.2)	7
Unknown	82 (3.7)	7
No growth	40 (1.8)	5

Adapted from the Myriam-webster dictionary, clinical features were characterised as observable and diagnosable symptoms [53] that may occur as a consequence of SI. Here we can consider determinants as a priori red flags leading to the development of SI, and the a posteriori as the the signs symptoms and clinical features that are present after the onset of SI.

In total there were 46 items, 23 determinants and 23 clinical features. For convenience and ease of use from a clinical perspective, the data were aggregated into relevant groups. The most frequently reported determinants were Diabetes (18%), Intravenous drug use (9%) and Surgery (6%) and the most frequently reported clinical features were Spinal Pain (72%), fever (55%) and Neurological Dysfunction (33%). Other salient red flags were immunosuppression (3%), invasive procedures (2%), corticosteriod use (2%), and history of TB (1%).

The pathogens reported were Bacterial (*n* = 1665), Fungal (*n* = 12), mixed organism (*n* = 27) and unknown growth (*n* = 82), with the most common microorganisms being *Staphylococcus aureus* (27%; *n* = 598)*, Mycobacterium tuberculosis* (12%; *n* = 262) and *Brucella* (9%; *n* = 210) (Table 3). There were no reports found on the other potential pathogenic causes of SI i.e. Viral, Prionic or Parasitic

## Discussion

Based on our initial search there were two potential studies evaluating the diagnostic accuracy of red flags for SI. However, just based on these two studies it was not possible to examine the diagnostic accuracy of red flags for SI, as one study combined the sensitivity and specificity for fever, spine pain, and neurologic deficits (classic triad) [54], while the other study, due to the low prevalence of SI, failed to gauge the sensitivity and specificity of red flags for SI [35]. This is consistent with the findings of Verhagen et al. in a recent systematic review looking at the red flags reported in current low back pain guidelines [3]. Spinal Infection is a relatively rare condition with an incidence of 0.2–2.4 cases per 100,000 annually in western societies [55, 56], this low incidence rate makes it virtually impossible to design a prospective diagnostic study [30, 32], resulting in a large body of retrospective case series and case report studies with no diagnostic accuracy data.

The majority of studies reviewed were carried out in the secondary care setting with a small number (5%) in the primary care setting [15, 57]. This underlines how difficult it is to identify SI in the early stages as the onset is insidious, there can be a protracted prodromal period and the clinical features are not highly specific, therefore many patients are likely to have been referred from primary care to secondary care for a diagnosis.

### Determinants

Red flags currently used in clinical practice that are considered specific to infection are; the use of corticosteroids, or immunosuppressant therapy, Intravenous drug abuse, past history of TB and fever [58, 59]. These, with the exception of fever, are all related to determinants rather than clinical signs of infection. The results of this paper concur and all of these determinants are reported, however, the most reported determinant was diabetes which featured in 30 papers. Although diabetes appeared as a determinant in a large number of papers it is interesting that it was only reported in 18% of patients. The review highlights that a number of morbidities such as diabetes, cancer, and HIV are among those conditions associated with immunosuppression which can consequently result in a suscebtibility to infection. Determinants such as corticosteroid use and alcohol abuse can also lead to a risk of immunodeficiency [60–63]. Also of note is that diseases such as rheumatoid arthritis and cancer sufferers treated with medications known to cause immunodeficiency (e.g. Disease-modifying anti-rheumatic drugs (DMARDS), steroids) are also at risk of SI. Determinants for SI (including intravenous drug use, diabetes and cancer) have high sensitivity (98%) and negative predictive value (99%) making them a better predictor of SI than clinical features such as the ‘classic triad’ (spinal pain, fever and neurological dysfunction) [54].

Spinal surgery and invasive procedures are regarded as having a high risk of infection with a rate of 1–4% reported elsewhere [64]. However, in this review, surgery and invasive procedures were reported as 6 and 2%, respectively. With procedural-induced SI, there are modifiable and non-modifiable determinants, such as sterility and immunological state [65–67]. Furthermore, post-procedural infection rate is highly linked to the invasiveness and complexity of the procedure carried out and the instrumentation used [68].

The overall mean age for those presenting with SI in this review was 55 years; previous research supports the notion that SI is a disease of older people [69, 70]. Amadoru et al. specifically looked at differences in presentation and outcomes between younger and older patients with SI [13]. Their findings suggested that older patients with SI present with fewer typical clinical features including fever and rigour and tend to seek medical advice later than younger patients. This could explain why age is a predictor for medical treatment failure [71]. However, the older population are also more likely to suffer from multi-morbidities which can suppress the immune system making them more susceptible to SI. A past history of TB is a question routinely asked by clinicians but it is only mentioned in one paper in this review [72]. Whilst Spinal TB is rare it is on the rise. The incidence of TB is dependent on birthplace and environmental factors, for example, born in countries with a high burden of TB, social conditions such as living in urban overcrowded conditions and homelessness. However, in this review, the social conditions reported related to rural living and occupational exposure [73].

### Clinical features

The most commonly reported features of SI reported in this review were back pain, fever and neurological dysfunction which has been described elsewhere as the classic triad [54]. The classic triad is the hallmark of the SI but reliance on patients presenting with these features is likely to result in missed cases or diagnosis in the late stages as not all patients will present with all three features. The most common reported symptom in this review is that of back pain (71%), however, back pain is usually a benign condition with a prevalence of 80% in the general population [74], which can present a diagnostic challenge for clinicians and in isolation it does not aid in identifying SI. In primary care, it is often back pain symptoms that prompt patients to seek help in the first place. Equally neurological symptoms are also reported but are also prevalent in the general population. SI is a progressively worsening disease with neurological deficit occurring in the later stages, whereas in the general population the expectation would be that after a period of time these neurological symptoms would resolve. Fever was the second most reported clinical feature of infection in this review. However, it was only prevalent in 55% of patients suggesting that a lack of fever cannot rule out SI and clinicians should not necessarily be reassured by its absence. These findings are consistent with other literature [70, 75–77], with Davis et al. reporting the ‘classic triad’ to be 8% sensitive and 99% specific [54].

### Clinical presentations

Overall*, Staphylococcus aureus* was the most reported cause of SI. Whereas Viral, Prionic and Parasitic causative pathogens were not reported at all. This is comparable with the wider literature as other systematic reviews also report bacterial SI, in particular, *Staphylococcus aureus,* to be the dominant causative SI agent [70, 76]. Though rare and not reported in this review, Viral and Parasitic SI do exist [78, 79]. In addition to this, Prionic diseases are even rarer, they are diseases of the nervous system affecting both humans and animals, there is no evidence of them affecting the musculoskeletal system, in particular, the spine [80].

### Diagnosis

Despite their low sensitivity (between 43 and 75%) [81], plain radiographs have traditionally been considered as the first step for assessing vertebral destruction [82, 83]. CT and MRI scans are highly sensitive, the results in this review show that CT and MRI scans were the most accurate radiological tests for diagnosing SI. Though, it is suggested that CT scans fail to properly detect epidural abscesses and spinal cord lesions, as such they are the procedure of choice only when MRI cannot be performed [82]. Finally, due to its diagnostic accuracy and non-invasive nature, MRI is the imaging of choice when investigating suspected SI, in particular, the early stages. MRI sensitivity, specificity, and accuracy for detecting SI are reported as 96, 92, and 94%, respectively [83–86]. Nevertheless, advanced diagnostic tools such as MRI and CT scans are costly and less available in primary care and low-income settings [87, 88], using them as the first diagnostic step for back pain patients is inefficient and wasteful. Therefore, it is important and cost-effective for clinicians to be equipped with the red flags to screen and decide whether further diagnostic tests are needed [87]. In one of the studies reviewed, Ferrari looked at the outcome of patients with spinal pain who were not referred to advanced imaging unless they presented with red flags, he found that using red flags as the threshold before ordering advanced imaging has a low threshold for missing serious spinal pathology [31].

### Limitations

To the author’s knowledge, this is the first review to investigate the use of red flags to identify SI. This review has identified gaps in research and knowledge and recommends future areas of research [89]. Our findings should be understood in the context of some limitations. Firstly, as we were not able to identify studies presenting diagnostic accuracy of red flags for spinal infection, as a result, we presented a descriptive review of the characteristics of people with spinal infection. As there was no control group to facilitate comparison of different determinants and clinical features, we do not know whether many of these features were more common in people with spinal infection compared to those without. As these results only tell us what features are common in people with spinal infection, they cannot be relied upon for diagnosis. Secondly, most of the studies included in this review were from high income (HI) countries, yet the global burden of infections such as Spinal TB falls mostly on low income and low middle-income countries (LMI); countries where TB, Brucellosis and HIV are endemic [90]. This might suggest selection bias (Table 1) but may also be as a result of underdiagnoses and underreporting of SI in LMI countries. LMI countries tend to have weak public health infrastructure and poor health-care research capacity compared to HI countries [91]. Furthermore, the disparity in the burden of disease in LMI countries and HI means efforts and resources are prioritised to different diseases [92]. Though the potential for publication bias must not be ruled out, there is also some research alluding to the possibility of an editorial bias against the academic output from low-income countries [93, 94].

### Recommendations

The current evidence surrounding red flags for SI remains of low quality and clinical features alone should not be relied upon to identify SI. When a patient arrives in clinic understanding determinants of possible SI should initially be considered. These risks include immunosuppression due to co-morbidities or drug use and environmental factors (surgery and social circumstances). The prevalence of these determinants combined with the presentation of clinical features of spinal pain with possible neurological dysfunction and fever should lead to a lowered threshold (or heightened index of suspicion) to further investigate and diagnostic tests should be performed to rule out SI. MRI is the imaging technique of choice when investigating suspected SI. The authors, therefore, suggest grouping red flags into a priori and a posteriori. The a priori red flags being the comorbidities and determinants that may be present leading to the development of SI, and the a posteriori being the signs symptoms and clinical features that are present after the onset of SI. Temporally stratifying red flags in this manner will aid the clinician in building a picture based on both potential risk of SI and presentation consistent with SI.

## Conclusion

Due to the paucity of literature on red flags for spinal infection, it was not possible to assess the diagnostic accuracy of red flags for spinal infection, as such, the authors conducted a descriptive review reporting the characteristics of those presenting with spinal infection was carried out. Based on the reviewed studies, spinal infection was common in those who had conditions associated with immunosuppression. Additionally, the most frequently reported clinical feature was the classic triad of spinal pain, fever and neurological dysfunction.

## Data Availability

All data generated or analysed during this study are included in this published article.

## Abbreviations

CRP: C-Reactive Protein (CRP)
CT: Computed Tomography
DMARDS: Disease-Modifying Anti-rheumatic Drugs
ESR: Erythrocyte Sedimentation Rate (ESR)
HIV: Human Immunodeficiency Virus
MPS: Medical Protection Society
MRI: Magnetic Resonance Imaging scan
ND: No data
NHS: National Health Service
NNHLBI: National Heart, Lung and blood institute
PROSPERO: Prospective Register of Systematic Reviews
SI: Spinal Infection
TB: Tuberculosis
WBC: World Bank Classification
WHO: World Health Organisation
